# Canine leptospirosis in Canada, test-positive proportion and risk factors (2009 to 2018): A cross-sectional study

**DOI:** 10.1371/journal.pone.0270313

**Published:** 2022-06-24

**Authors:** Jason W. Stull, Michelle Evason, J. Scott Weese, Jenny Yu, Donald Szlosek, Amanda M. Smith

**Affiliations:** 1 Department of Health Management, Atlantic Veterinary College, University of Prince Edward Island, Charlottetown, PE, Canada; 2 Department of Veterinary Preventive Medicine, College of Veterinary Medicine, The Ohio State University, Columbus, Ohio, United States of America; 3 Department of Companion Animals, Atlantic Veterinary College, University of Prince Edward Island, Charlottetown, PE, Canada; 4 Department of Pathobiology, Ontario Veterinary College, University of Guelph, Guelph, Canada; 5 IDEXX Laboratories, Inc., Wetherby, United Kingdom; 6 IDEXX Laboratories, Inc., Westbrook, ME, United States of America; 7 Baltimore Department of Health, Baltimore, MD, United States of America; Animal Health Centre, CANADA

## Abstract

Over the past decade, there has been an apparent increased frequency and widened distribution of canine leptospirosis in Canada, however, this has been minimally investigated. Availability and clinical uptake of *Leptospira* polymerase chain reaction (PCR)-based testing of dogs in Canada may provide important insight into the epidemiology of this canine and zoonotic infectious disease. Study objectives were to evaluate clinical canine *Leptospira* PCR test results from a large commercial laboratory to determine temporal and spatial distribution in Canada and identify dog, geographic and temporal risk factors for test-positive dogs. This cross-sectional study analyzed data obtained from IDEXX Laboratories, Inc. on 10,437 canine *Leptospira* PCR tests (blood and/or urine) submitted by Canada-based veterinarians (July 2009 to May 2018). Multivariable logistic regression was used to identify risk factors for test-positive dogs. Test-positive proportion varied widely annually (4.8–14.0%) and by location. Provinces with the highest test-positive proportion over the study period were Nova Scotia (18.5%) and Ontario (9.6%), with the prairie provinces (Manitoba and Alberta combined) having the lowest proportion (1.0%); the northern territories could not be evaluated due to limited testing. In the final model, dog age, sex, breed, month, and year test performed, and location (urban/rural, province) of the practice submitting the sample were significant predictors of a positive *Leptospira* PCR test. Dogs less than one year of age (OR = 2.1; 95% CI: 1.6–2.9), male sex (OR = 1.3; 1.1–1.5), toy breed (OR = 3.3; 2.5–4.4), and samples submitted from an urban practice (OR = 1.3; 1.0–1.8) had the greatest odds of a positive *Leptospira* PCR test as compared to referent groups. Significant two-way interactions between province-month and year-month highlight the complex spatial and temporal influences on leptospirosis occurrence in this region. Our work suggests a high incidence of canine leptospirosis regionally within Canada. Identifiable dog and location factors may assist in future targeted prevention efforts.

## Introduction

Leptospirosis is a globally important zoonotic disease. Spread primarily by the urine of animal host species, historically leptospirosis was predominately diagnosed in dogs that had rural lifestyles (e.g., live on livestock farms, take part in rural outdoor activities such as field trials). The epidemiology of canine leptospirosis has evolved over recent years, identifying five serovars (each with varying reservoir host species) that appear to be most important for canine health in North America. Peridomestic wildlife species (e.g., rodents, raccoons), as well as dogs are reservoirs of key *Leptospira* serovars, supporting the increased recognition of leptospirosis as an important disease of dogs residing in strictly urban environments. Different *Leptospira* serovars are present in different areas of North America, likely reflecting regional variations in the epidemiology of the disease [1–5]. The incidence and seroprevalence of *Leptospira* spp. in dogs appear to be increasing, particularly in North America [1–5]. Clinical disease in dogs may be severe, and therapy frequently entails costly treatment and long-term monitoring [2, 6]. Further, infected dogs may serve as a source of infection for other animals and people, and common environmental exposure may allow dogs to serve as a sentinel for human risk [2, 7]. Despite the importance of leptospirosis for canine health, the epidemiology of this disease in dogs is poorly understood. This knowledge gap is particularly evident in Canada, where canine leptospirosis has been minimally studied, with existing studies limited by region and date [1, 8, 9]. Further, recent anecdotal data suggest increased disease incidence in eastern and Atlantic Canada, with a large, suspected outbreak in Nova Scotia, Canada in 2017 [10].

Multiple diagnostic methods have been developed to identify *Leptospira*-infected dogs. Unfortunately, diagnosis can be challenging with some testing methodologies based on antibody response, making it difficult to differentiate clinical disease from prior exposure or vaccination. Most prior studies of leptospirosis in dogs have used such antibody-based tests (e.g., microscopic agglutination test; MAT) [1, 3–5, 11]. In recent years, polymerase chain reaction (PCR) leptospirosis testing has become increasingly used in clinical veterinary medicine. The PCR test may reduce the interpretation challenges commonly encountered with antibody-based tests, extending such benefits to population-based studies of the disease. At present, diagnosis is typically confirmed through consistent clinical signs, suggestive clinicopathologic changes (thrombocytopenia, renal and/or liver enzyme elevations, dilute urine), response to appropriate antimicrobials, and either PCR (urine, blood, or both) and/or serology testing (ideally, paired acute and convalescent microscopic agglutination test (MAT)) serology or in-clinic ELISA (IgG or IgM) [2, 6, 12].

Prevention of disease is most effectively accomplished through avoidance of contaminated environments. However, the ability to completely avoid contaminated areas is challenging and typically impractical. *Leptospira* vaccination is generally considered non-core, and administration relies on practitioners’ level of awareness and ability to make an appropriate risk assessment of the dog based on location, lifestyle, and other factors [13].

Given the scarcity of Canadian specific publications on leptospirosis, anecdotal information regarding increased disease incidence in eastern and Atlantic Canada, and importance of reliable data to inform dog owner and veterinarian risk assessment and targeted prevention, there is a clear need for further work in this area. Similar habitat risks and approaches to prevention may be applicable to dog and human disease, and dogs may serve as sentinels for human health risks [7]. Thus, addressing research gaps for dogs may have applications to human leptospirosis prevention.

The objectives of our study were to evaluate a clinical dataset of canine *Leptospira* PCR positive test results and determine temporal (month, annual) and spatial distribution in Canada, and to identify dog, geographic, and temporal risk factors for PCR-positive test dogs.

## Material and methods

This was a cross-sectional study that used 10,437 PCR test results for canine leptospirosis. Tests were submitted between July 1, 2009 and May 1, 2018 to IDEXX Laboratories, Inc. Data were obtained from the reported results of routine clinical tests (IDEXX Real-PCR® Test) performed on blood and/or urine samples from dogs submitted from veterinary clinics in Canada. Permission to access and use data was obtained from IDEXX Laboratories, Inc. The PCR test used has been validated in dogs, with reported high sensitivity and specificity (92% and 99%, respectively using MAT as the gold standard) [14]. In summary, the PCR test is based on IDEXX’s proprietary real-time PCR oligonucleotides (IDEXX Laboratories, Westbrook, Maine). Hap-1 gene sequences were aligned, a region was selected for primer and hydrolysis probe design, and real-time PCR was run with standard primer and probe concentrations using the Roche LightCycler 480 Probes Master mastermix (Version 3.0, Applied Biosystems). The test detects *Leptospira* spp. DNA from only the recognized pathogenic strains due to the presence of the hap1 gene, including *L*. *interrogans* and *L*. *kirschneri* [15]. The same test was used over the study period.

All dogs were assumed to be client-owned, and diagnostics and treatments were at the discretion of the client. Specific clinical data were unavailable, but it was presumed that most dogs were tested due to presence of clinical signs suggestive of leptospirosis. Data were available in electronic database format, with data on month and year test performed, dog signalment (age in days, sex/reproductive status, breed), Canada Post forward sortation area (FSA; first three postal code characters) for the submitting veterinary clinic, test result, and dog unique identifier. Dog unique identifiers were created by IDEXX Laboratories, Inc. based on a combination of dog name, owner name and clinic ID and verified by study author (JWS) for entries with the same unique identifier based on signalment and FSA information. Postal code for the dog’s residence and vaccination status were unknown. Repeat entries were removed. An entry was considered a repeat if the same dog (based on unique identifier, signalment, and FSA) was tested more than once in a given calendar year or, for December and January entries, spanning two calendar years. If the test outcomes for a set of repeat entries were the same, the most recent entry was retained in the dataset and additional entries were removed. If the test outcomes differed for a set of repeat entries, a single positive (most recent) entry was retained. From these data, variables were derived for the dog’s age in years at the time of testing (≤ 1.0, 1.1–4.0, 4.1–7.9, ≥ 8.0), AKC breed group (sporting, herding, hound, non-sporting, terrier, toy, working, mixed; based on breed listed, if more than one breed listed, then categorized as mixed), month when testing was performed (Jan-Feb, Mar-Apr, May-Jun, Jul-Aug, Sep-Oct, Nov-Dec), and submitting veterinary location (rural/urban based on second character in FSA of submitting veterinary clinic), and province/region [British Columbia (BC), Ontario (ON), Quebec (QC), Nova Scotia (NS), Atlantic Canada [Prince Edward Island (PE), New Brunswick (NB), Newfoundland and Labrador (NL)], prairie provinces [Alberta (AB), Manitoba (MB)] [16, 17]. Although geographically NS is included within Atlantic Canada, it was separated for analysis due to anticipated differences in the epidemiology of canine leptospirosis between these regions.

### Data maps and analysis

#### Data mapping

To visualize the spatial distribution of testing and positive canine *Leptospira* test results, the frequency of tests performed, frequency of test-positive dogs, and test-positive proportion of dogs were separately mapped by FSA for all years combined using FSA boundary files from Statistics Canada and ArcGIS version 10.2.2 (Environmental Systems Research Institute) [18, 19]. Calculating incidence-type measures is challenging with companion animals, as the owned canine population in Canada is unknown. In this circumstance, the human population was used as a proxy for the canine population; we calculated this measure by dividing the number of positive canine *Leptospira* PCR tests over the study period for a given FSA by the 2016 human census population for that FSA (reported as test-positive dogs per 100,000 people). Test-positive dogs per 100,000 people by FSA was mapped as described above. Potential ‘hot spots’ of canine *Leptospira* in Canada were identified visually as areas (FSAs) with relatively increased number of cases, increased test-positive proportion, and increased number of cases per human capita as compared to surrounding FSAs.

#### Data analysis

Test-positive proportion of leptospirosis at the dog-level was calculated overall and for subgroups (province/region, year, month) by dividing the number of positive canine *Leptospira* PCR tests by total number of tests. Ninety-five percent Clopper-Pearson confidence intervals were calculated. Years for which only partial year data were available (2009 and 2018), were excluded from annual descriptive statistics (test-positive proportion) but were included in all model building.

The association between dog, temporal, and spatial variables and a positive *Leptospira* PCR test was explored using logistic regression models. The main outcome of interest was a positive canine *Leptospira* PCR test. Descriptive statistics, Odds Ratios (OR), and 95% confidence intervals (CI) for the ORs were calculated for all variables.

Univariable logistic regression models were built and variables with a likelihood ratio test *P*-value < 0.2 were eligible to be tested for inclusion in the final multivariable model. Spearman’s rank correlation (Phi coefficient for two dichotomous variables) was performed between all predictors eligible for multivariable analysis. When predictors were highly correlated (correlation coefficient ≥ |0.80|), one variable was retained based on perceived importance/relevance for drawing conclusions from the analysis. A final multivariable logistic regression model was built using a backwards stepwise approach. Confounding was assessed when removing variables from the multivariable model. Variables were kept in the model as confounders if their removal changed the coefficients of one or more retained terms by ≥20%. Statistical significance was based on a likelihood ratio test *P*-value < 0.05. Biologically relevant 2-way interactions between variables retained in the final multivariable model were assessed for significance using a likelihood ratio test. Predictive probabilities and associated 95% CIs for a positive test result were graphed to visualize interaction terms. Model fit was assessed with the Hosmer–Lemeshow goodness of fit test. Stata 16 (StataCorp, College Station TX) was used for analysis.

## Results

A total of 19,066 PCR test results were available over the study timeframe. Removal of 8,629 repeat entries was performed, the vast majority of which (7,882; 91%) were exact repeats except for sample source (urine, blood), resulting in 10,437 *Leptospira* PCR test results used in the analyses. Most records (8,454; 81%) were complete, with AKC breed group being the most frequently missing data element (8,807; 84% present) (Table 1).

**Table 1:** Proportion PCR-test positive and univariable and multivariable logistic regression models for canine *Leptospira* PCR tests in Canada (2009–2018).

Variable	N (%)	Proportion PCR Positive (95% CI)	Univariable Models		Multivariable Model	
			Odds Ratio (95% CI)	*P*-value	Odds Ratio (95% CI)	*P*-value
**Overall**	10,437	8.4 (7.9, 9.0)				
**Dog age (years)**	10,309			**<0.0001** ^#^		**<0.0001** ^#^
≤ 1.0	687 (6.7)	13.0 (10.5, 15.7)	2.1 (1.7, 2.7)	<0.001	2.1 (1.6, 2.9)	<0.001
1.1–4.0	1,882 (18.3)	10.1 (8.8, 11.5)	1.6 (1.3, 1.9)	<0.001	1.6 (1.3, 2.0)	<0.001
4.1–7.9	3,263 (31.7)	9.2 (8.2, 10.2)	1.4 (1.2, 1.7)	<0.001	1.5 (1.2, 1.8)	<0.001
≥ 8.0	4,477 (43.4)	6.5 (5.8, 7.3)	Reference		Reference	
**Dog sex**	10,363			**0.001** ^#^		**0.001** ^#^
Female	4,959 (47.9)	7.2 (6.5, 7.9)	Reference		Reference	
Male	5,404 (52.1)	9.6 (8.8, 10.4)	1.4 (1.2, 1.6)		1.3 (1.1, 1.5)	
**AKC Breed Group**	8,807			**<0.0001** ^#^		**<0.0001** ^#^
Sporting	1,879 (21.3)	4.4 (3.5, 5.4)	Reference		Reference	
Herding	969 (11.0)	8.9 (7.2, 10.8)	2.1 (1.6, 2.9)	<0.001	2.0 (1.4, 2.8)	<0.001
Hound	423 (4.8)	7.8 (5.4, 10.8)	1.9 (1.2, 2.8)	0.004	1.8 (1.1, 2.8)	0.010
Mixed	1,454 (16.5)	9.1 (7.7, 10.7)	2.2 (1.7, 2.9)	<0.001	2.3 (1.7, 3.1)	<0.001
Non-sporting	797 (9.1)	8.3 (6.5, 10.4)	2.0 (1.4, 2.8)	<0.001	1.9 (1.3, 2.7)	<0.001
Terrier	840 (9.5)	9.4 (7.5, 11.6)	2.3 (1.7, 3.1)	<0.001	2.3 (1.7, 3.3)	<0.001
Toy	1,308 (14.9)	14.3 (12.4, 16.3)	3.7 (2.8, 4.8)	<0.001	3.3 (2.5, 4.4)	<0.001
Working	1,137 (12.9)	6.2 (4.9, 7.8)	1.5 (1.1, 2.0)	0.02	1.3 (1.0, 1.9)	0.1
**Month test performed** ^4^	10,437			**<0.0001** ^#^		**<0.0001** ^##^
Jan-Feb	1,406 (13.5)	2.6 (1.9, 3.6)	1.5 (0.9, 2.4)	0.1	NR	NR
Mar-Apr	1,630 (15.6)	1.8 (1.2, 2.5)	Reference		Reference	
May-Jun	1,577 (15.1)	3.2 (2.4, 4.2)	1.9 (1.2, 2.9)	0.009	NR	NR
Jul-Aug	1,578 (15.1)	5.1 (4.1, 6.3)	3.0 (1.9, 4.6)	<0.001	NR	NR
Sep-Oct	2,032 (19.5)	14.5 (13.0, 16.1)	9.4 (6.4, 13.8)	<0.001	NR	NR
Nov-Dec	2,214 (21.2)	17.5 (15.9, 19.1)	11.7 (8.0, 17.2)	<0.001	NR	NR
**Year test performed** ^4^	10,437			**<0.0001** ^#^		**<0.0001** ^##^
2009^3^	220 (2.1)	NR	1.6 (1.0, 2.6)	0.07	NR	NR
2010	223 (2.1)	5.4 (2.8, 9.2)	0.8 (0.4, 1.5)	0.5	NR	NR
2011	810 (7.8)	4.8 (3.4, 6.5)	0.7 (0.5, 1.1)	0.09	NR	NR
2012	893 (8.6)	5.0 (3.7, 6.7)	0.8 (0.5, 1.1)	0.1	NR	NR
2013	933 (8.9)	11.4 (9.4, 13.6)	1.8 (1.4, 2.4)	< 0.001	NR	NR
2014	1,175 (11.3)	6.9 (5.5, 8.5)	1.1 (0.8, 1.4)	0.7	NR	NR
2015	1,326 (12.7)	7.3 (6.0, 8.9)	1.1 (0.8, 1.5)	0.4	NR	NR
2016	1,505 (14.4)	6.6 (5.4, 8.0)	Reference		Reference	
2017	2,581 (24.7)	14.2 (12.9, 15.6)	2.4 (1.9, 3.0)	< 0.001	NR	NR
2018^3^	771 (7.4)	NR	0.2 (0.1, 0.4)	< 0.001	NR	NR
**Postal code designation** ^1^	10,419			**0.04**		**0.04**
Rural	1,189 (11.4)	6.6 (5.3, 8.2)	Reference		Reference	
Urban	9,230 (88.6)	8.7 (8.1, 9.3)	1.3 (1.1, 1.7)		1.3 (1.0, 1.8)	
**Province** ^1^ ^,^ ^2^ ^,^ ^4^	10,432			**<0.0001** ^#^		**<0.0001** ^##^
British Columbia	1,671 (16.0)	3.6 (2.8, 4.6)	3.8 (1.2, 12.1)	0.03	NR	NR
Ontario	6,711 (64.3)	9.6 (8.9, 10.3)	10.8 (3.5, 33.7)	<0.001	NR	NR
Quebec	1,101 (10.6)	5.8 (4.5, 7.4)	6.3 (2.0, 20.1)	0.002	NR	NR
Nova Scotia	562 (5.4)	18.5 (15.4, 22.0)	23.0 (7.3, 73.2)	<0.001	NR	NR
Atlantic Canada (excluding Nova Scotia)	80 (0.8)	5.0 (1.4, 12.3)	5.3 (1.2, 24.3)	0.03	NR	NR
Prairie Provinces	307 (2.9)	1.0 (0.2, 2.8)	Reference		Reference	
**Interactions** ^4^						
Province*Month					NR	**0.005**
Year*Month					NR	**0.03**

^1^Location based on veterinary facility that submitted sample for testing.

^2^Prairie Provinces = Alberta, Manitoba; Atlantic Canada = Prince Edward Island, New Brunswick, Newfoundland, and Labrador.

^3^Data only available for partial calendar year.

^4^Part of an interaction term; cannot be interpreted without simultaneous evaluation of relevant main effects and interaction term. See Figs 6 and 7.

NR–Not reported since data only available for partial calendar year or part of an interaction term.

^#^Overall *P*-value for the variable effect

^##^Test for combined effect of the variable including interaction term

**Fig 1:**
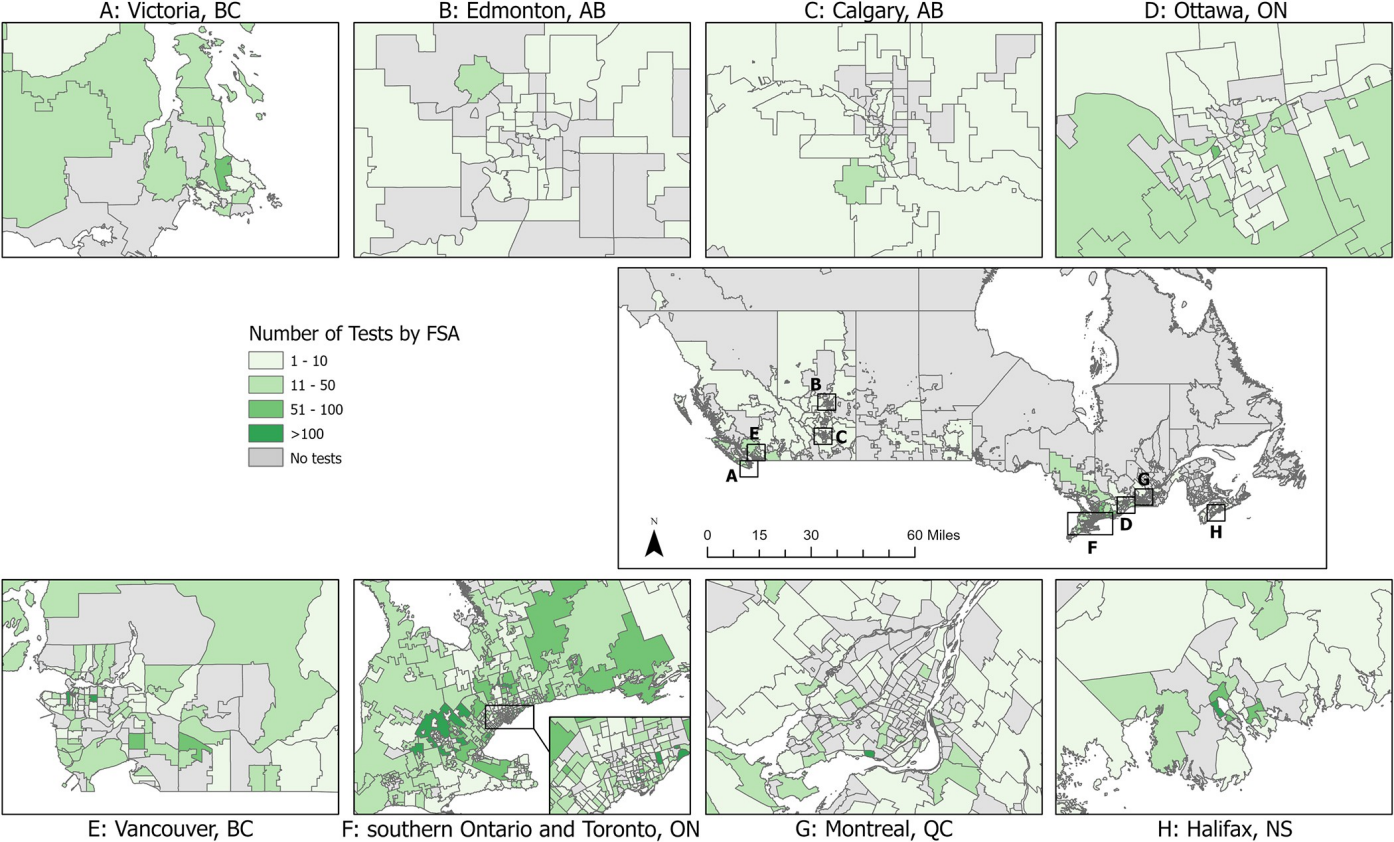
Number of submitted canine *Leptospira* PCR tests by forward sorting area (FSA) in Canada, 2009–2018. FSA for submitting veterinary location.

The population of dogs tested was 52% male and had a mean age of 6.9 years (SD 3.9; range 0.1–20). The number of PCR tests submitted increased each year (full calendar years 2010: 223; 2017: 2,581), with the greatest annual increase between 2010 (223) and 2011 (810) (263% positive change). Of the total 1,620 FSAs in Canada, samples were reported from 788 FSAs (48.6%; from which there were a median of 6 samples/FSA, range 1-283/FSA; Fig 1).

**Fig 2:**
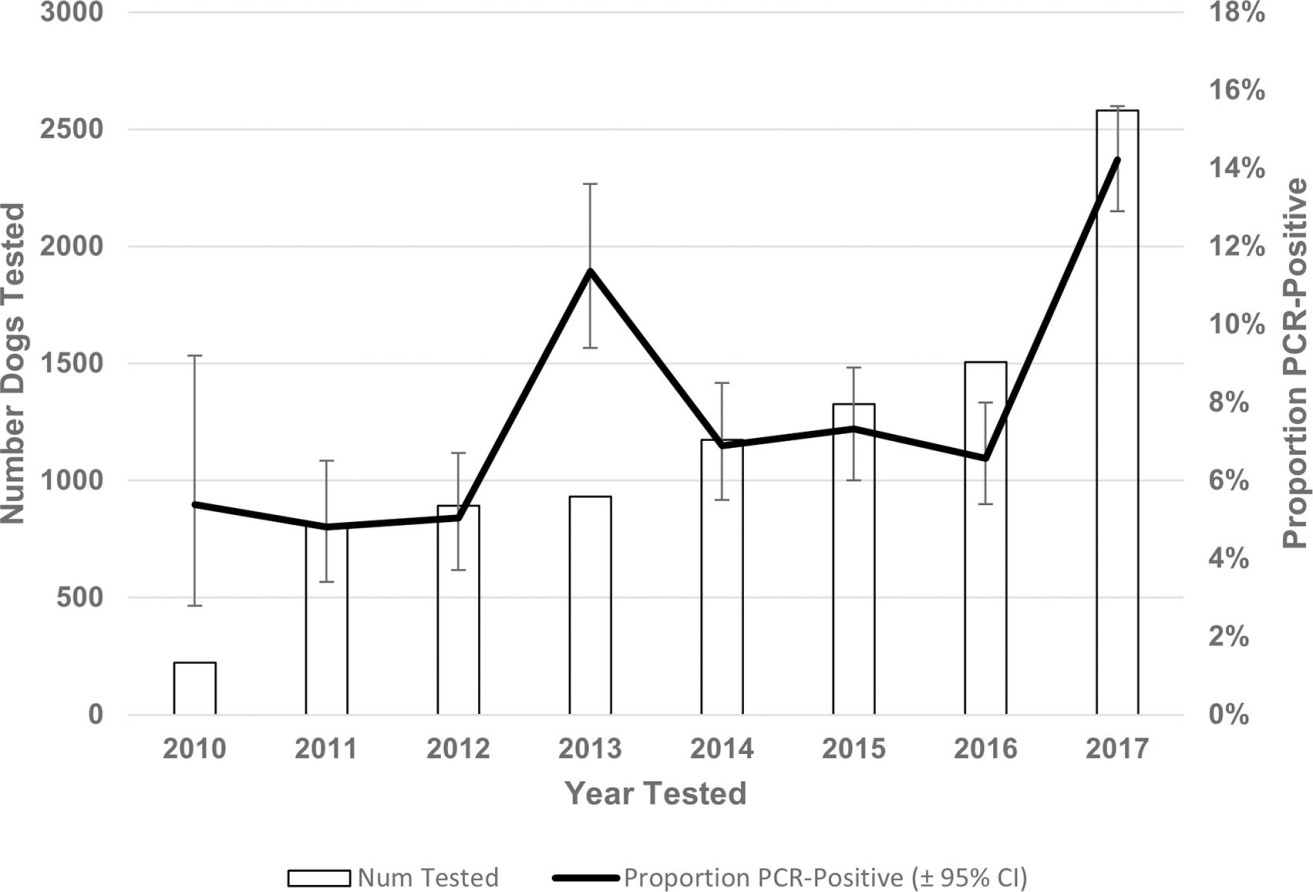
Canine *Leptospira* PCR testing in Canada (2010–2017) depicting annual number of dogs tested and proportion test-positive. Data from partial calendar years (2009 and 2018) excluded.

**Fig 3:**
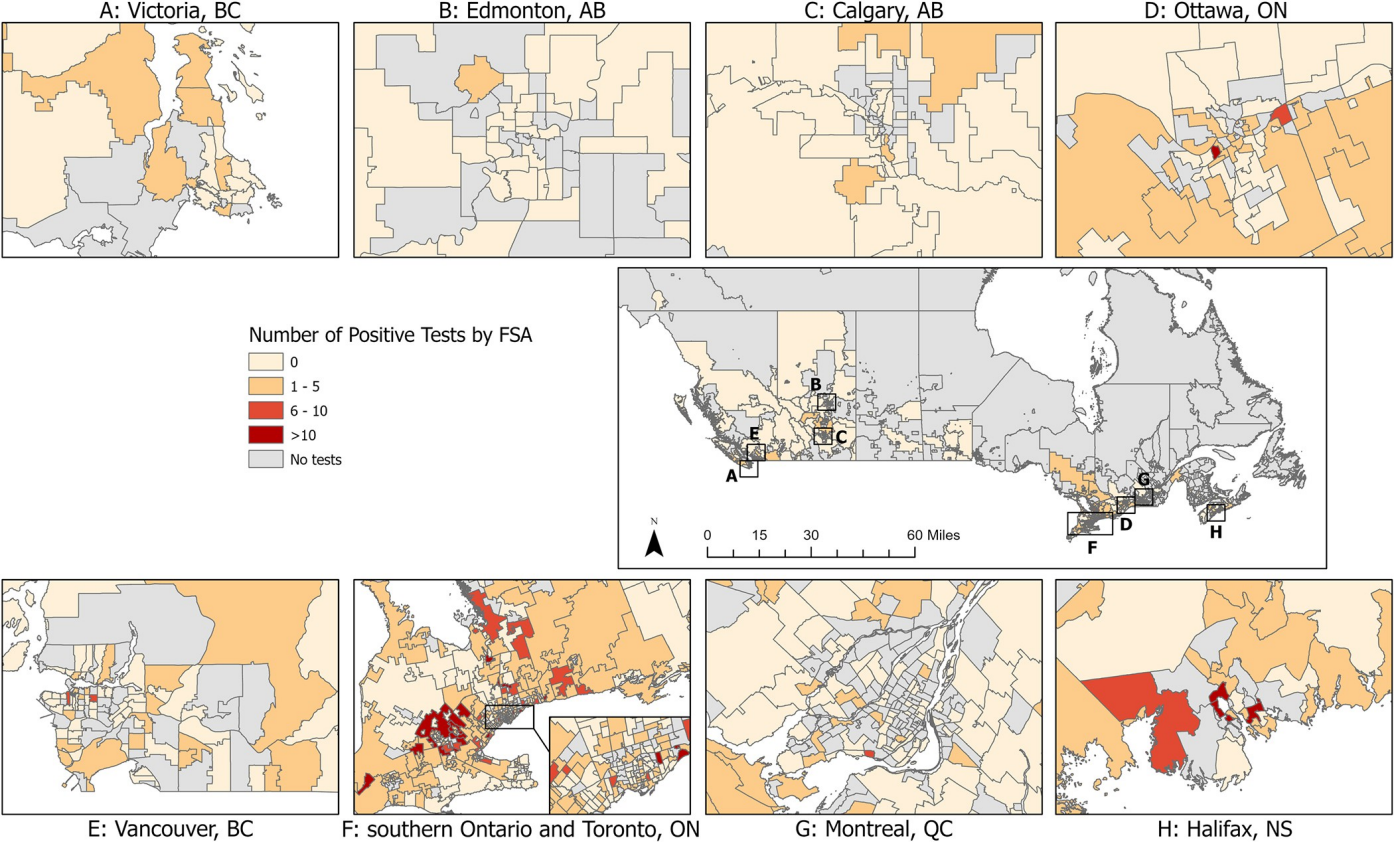
Number of positive canine *Leptospira* PCR tests by forward sorting area (FSA) in Canada, 2009–2018. FSA for submitting veterinary location.

**Fig 4:**
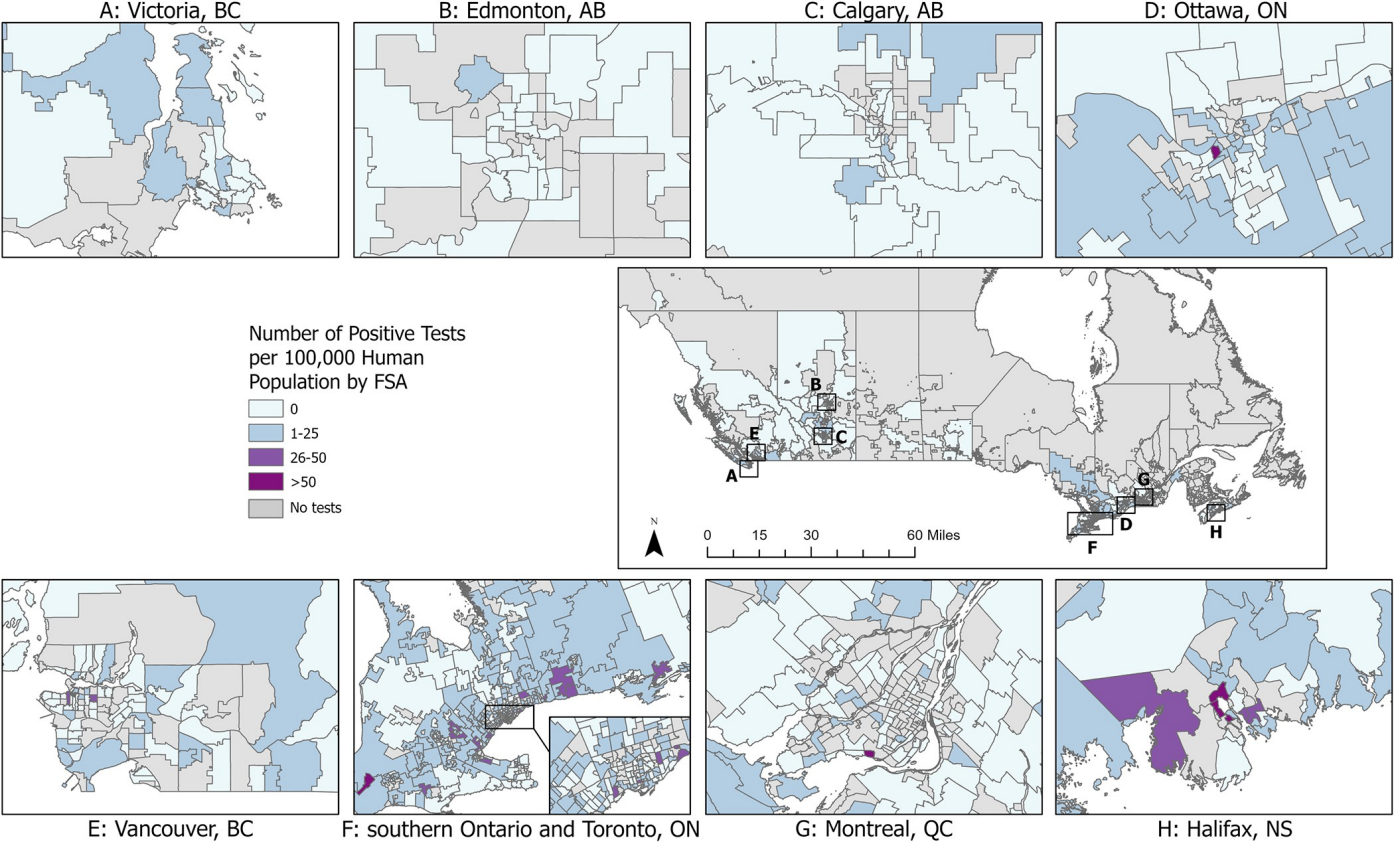
Number of positive canine *Leptospira* PCR tests per 100,000 human population by forward sorting area (FSA) in Canada, 2009–2018. FSA for submitting veterinary location.

**Fig 5:**
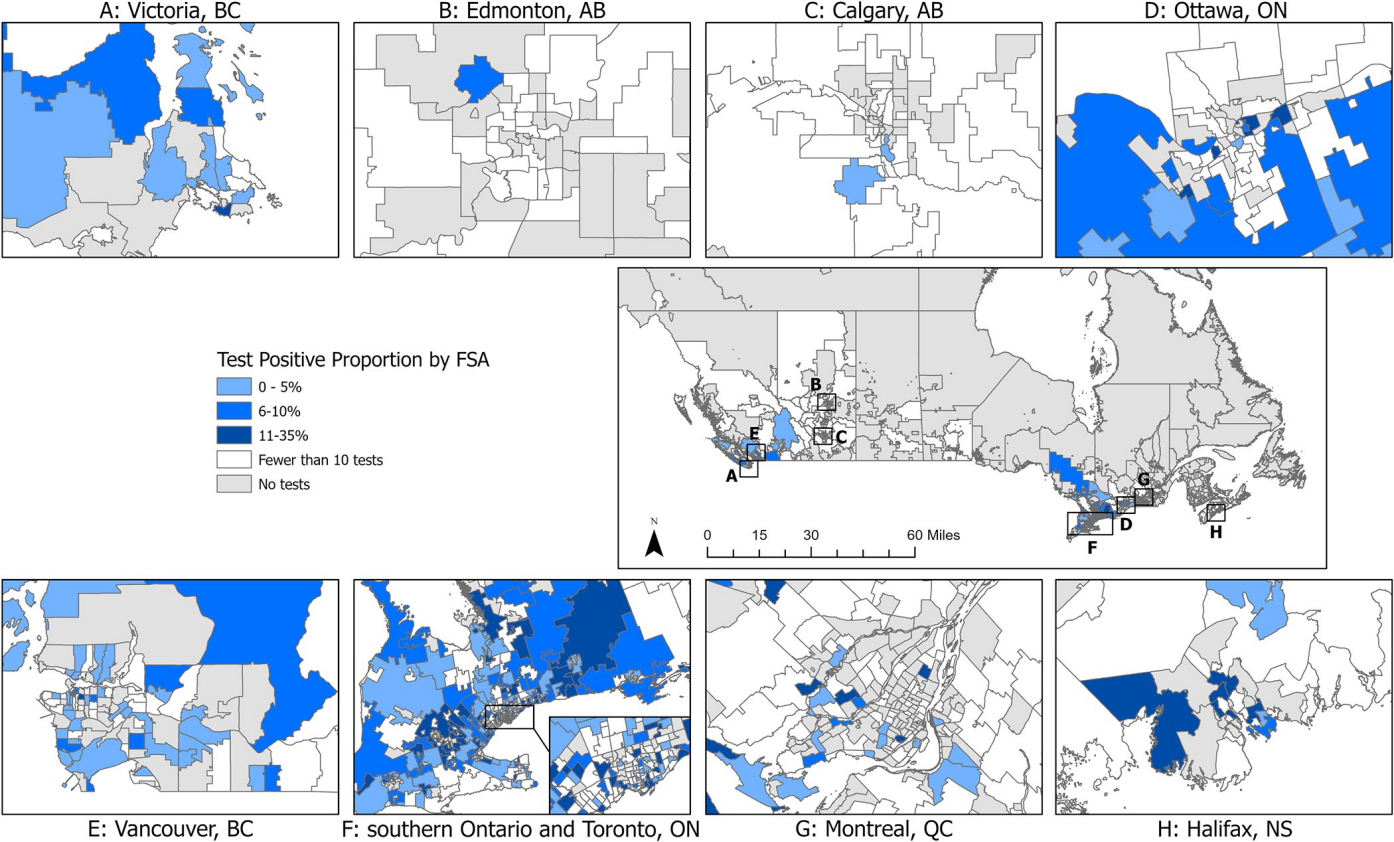
Test-positive proportion of canine *Leptospira* PCR tests by forward sorting area (FSA) in Canada, 2009–2018. FSA for submitting veterinary location.

One or more positive *Leptospira* PCR test results were reported from 880 dogs (8.4%; 95% CI: 7.9, 9.0). The proportion of test-positive dogs varied by year from 4.8% (39/810; 2011) to 14% (367/2,581; 2017) (Table 1, Fig 2). The number and proportion of test-positive dogs varied by Province from three (prairie provinces) to 645 (Ontario), 1.0% (prairie provinces) to 18.5% (Nova Scotia), respectively (Table 1). Three provinces were excluded from provincial comparisons due to low sampling: Northwest Territories (n = 0), Nunavut (n = 0), Yukon (n = 3, all negative results). Of the FSAs from which samples were submitted, 283 (35.9%) reported one or more positive dog (from which there were a median of 2 positive dogs/FSA, range 1-29/FSA). The frequency and test-positive proportion of *Leptospira*-positive dogs, and *Leptospira* test-positive dogs per 100,000 people varied by FSA (Figs 3–5). Overall, eastern and western metropolitan locations and their surrounding areas had the greatest values for these measures.

In the univariable analysis, dog signalment (age, sex, AKC breed group), location (province, rural/urban status), and time of testing (month, year) were significant predictors for a positive *Leptospira* test result (all *P* < 0.02) and retained in the final multivariable model (all *P* < 0.05; Table 1). In addition, the two-way interactions of province*month (odds of a positive test result reported in each month depended on the province where the test was performed) and year*month (odds of a positive test result reported in each month depended on the year the test was performed) were both significant predictors (each *P* < 0.04) when added to the main effects model and thus retained in the final model. The final model fit the data (Hosmer–Lemeshow *P* = 0.88).

In the multivariable model, younger dogs were at significantly increased odds of being *Leptospira*-positive as compared to elderly dogs (referent ≥ 8.0 yr), with dogs less than or equal to one year of age having the greatest odds of infection (OR = 2.1; 95% CI 1.6–2.9), followed by older dogs [1.1–4 years of age (OR = 1.6; 1.3–2.0), 4.1–8.0 years of age (OR = 1.5; 1.2–1.8)]. Male dogs were at significantly increased odds of being *Leptospira*-positive as compared to female dogs (OR = 1.3; 1.1–1.5) Amongst the AKC breed groups, all except working breeds were at significantly higher odds of being *Leptospira*-positive as compared to sporting breed dogs (referent), most pronounced with toy (OR = 3.3; 2.5–4.4), mixed (OR = 2.4; 1.7–3.2), and terrier breeds (OR = 2.3; 1.7–3.3). Dogs tested by veterinary practices in an urban setting were at significantly increased odds of testing positive for leptospirosis as compared to submissions from rural locations (OR = 1.3; 1.0–1.8).

**Fig 6:**
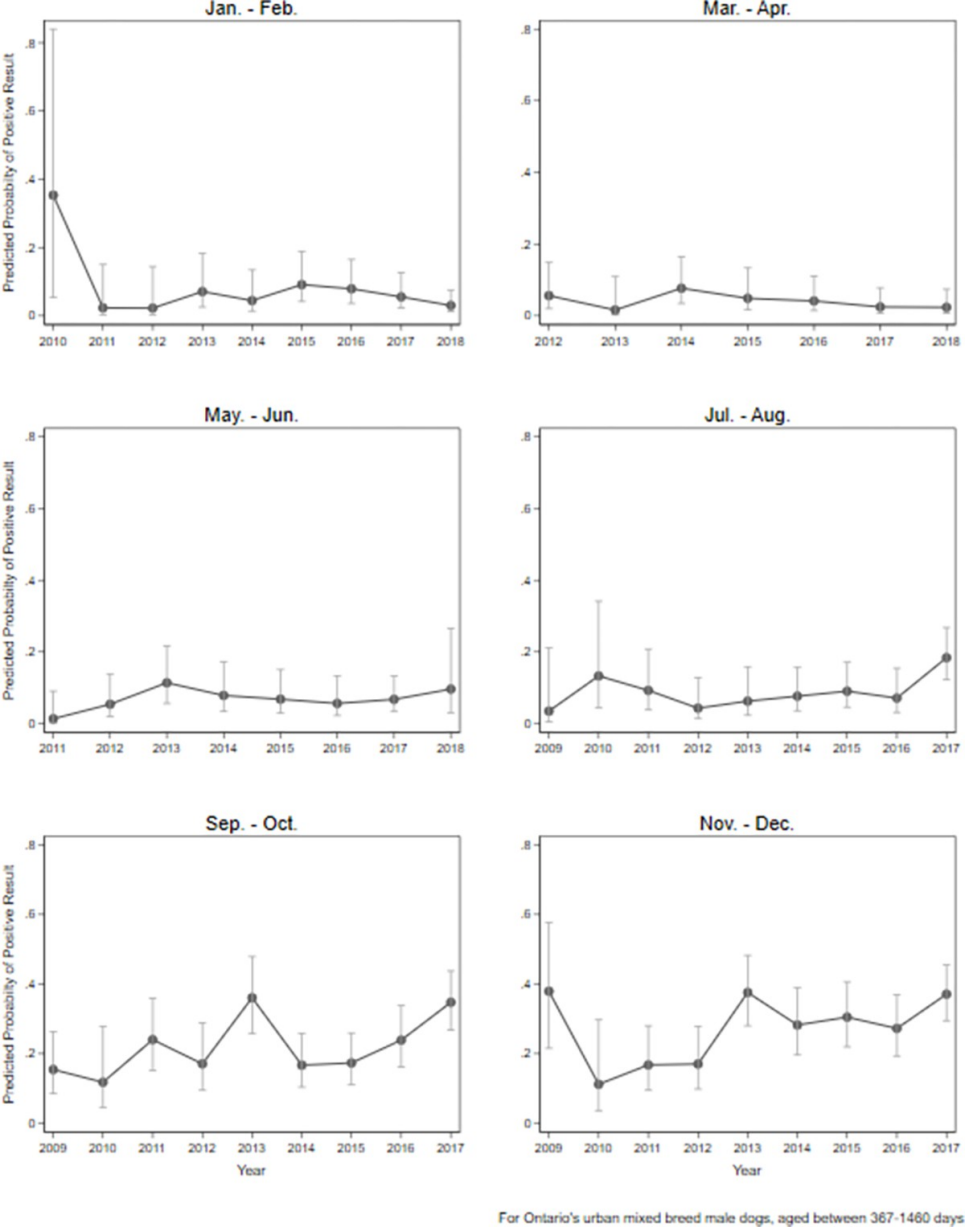
Margin plots of predicted probability (± 95% CI) of PCR-positive *Leptospira* test result in dogs by month and year of testing, Canada. Predicted probabilities based on an urban, mixed breed, 1–4-year-old dog tested in Ontario, Canada.

**Fig 7:**
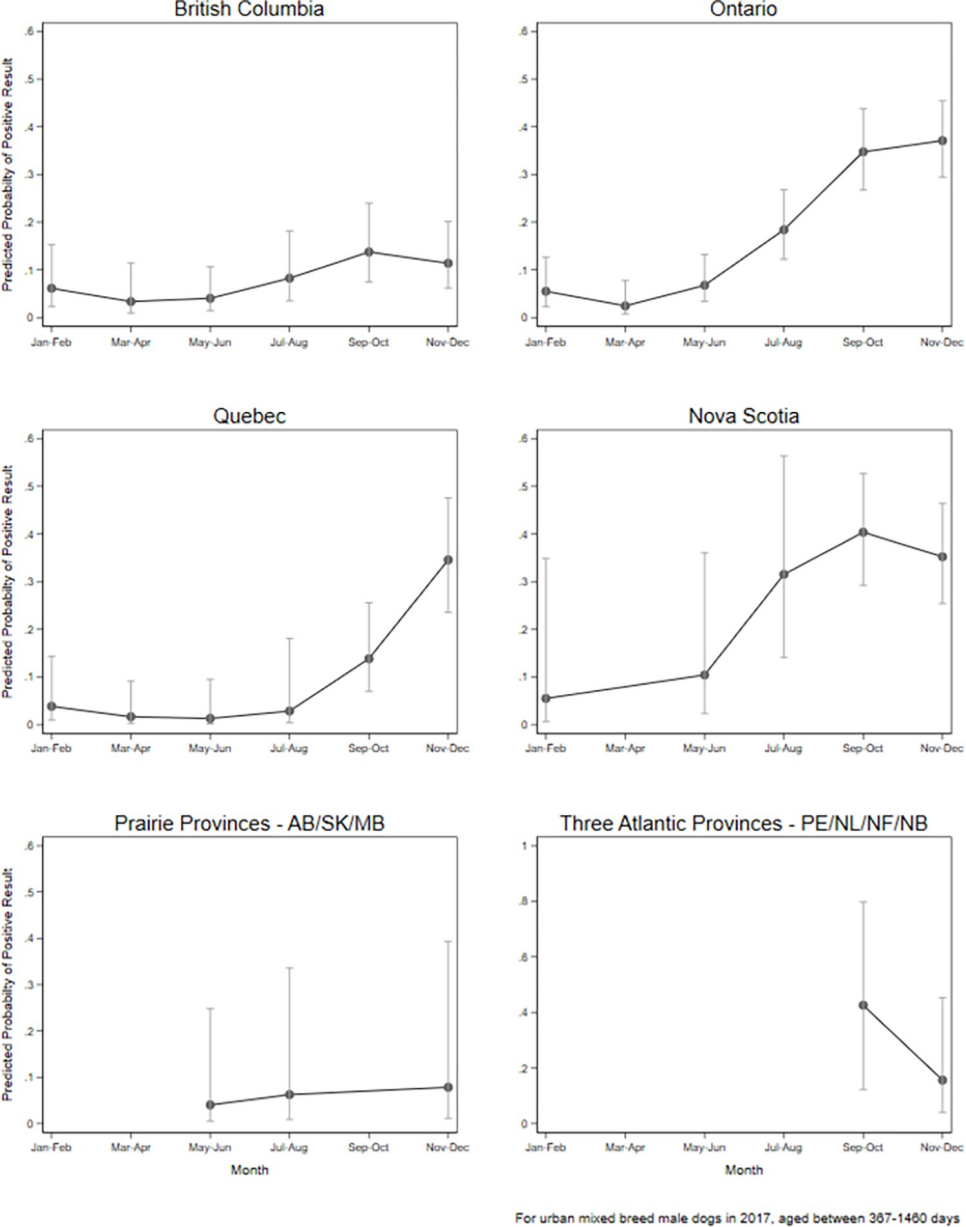
Margin plots of predicted probability (± 95% CI) of PCR-positive *Leptospira* test result in dogs by province and month of testing, Canada. Note: y-scale for the Atlantic Provinces is different than the others. Predicted probabilities based on an urban, mixed breed, 1–4-year-old dog tested in 2017.

Province, month, and year were included in interaction terms and therefore visualized with margins plots (Figs 6 and 7). From January through August, the predicted probabilities of dogs testing positive for *Leptospira* was relatively low (generally < 10%) with minimal annual deviations (exception July-August 2017), while in the latter half of the calendar year (Sept-Dec), the predicted probabilities were generally higher (> 10%) with more pronounced annual deviations (Fig 6). Differing effects of time of year (month) were noted among the provinces. Ontario, British Columbia, Quebec, and Nova Scotia revealed an increased predicted probability of dogs testing positive for *Leptospira* in the fall/winter (September-December; Fig 7). This was most pronounced in Ontario and Nova Scotia, which had the greatest peak predicted probabilities (~40%), while a mild increased peak predicted probability was noted in British Columbia. Limited data made it difficult to accurately predict the probabilities of dogs testing positive for *Leptospira* in the prairie and Atlantic provinces.

## Discussion

There have been few studies on canine leptospirosis in Canada [1, 8]. As such, the epidemiology of the disease in the country remains poorly defined and limited to a single geographical area (Ontario). In the United States, recent MAT-positive prevalence for canine leptospirosis was estimated to be 14% between 2000 and 2014 [11]. Another US study, evaluating canine *Leptospira* PCR tests submitted through a commercial diagnostic laboratory (2009 to 2016) found an overall test-positive proportion of 5.4% [20]. Our PCR-based work identified an overall Canadian canine leptospirosis test-positive proportion of 8.4%. While it is important to acknowledge that clinical data were not available for our study, preventing us from confirming that test-positive dogs were clinically affected leptospirosis cases, we presume that the results of the PCR testing likely reflect clinical disease. This is because *Leptospira* testing would be predominantly performed in dogs with signs of disease [21]. As the PCR test used only detects *Leptospira* spp. nucleic acid of pathogenic strains, a positive test result in a dog with clinical disease supports recent infection. The analysis of PCR-based data lessened challenges commonly observed with leptospirosis MAT interpretation (e.g., interference related to vaccination and exposure) [22].

Prior studies have consistently noted annual and geographic fluctuations in the occurrence of canine leptospirosis [11, 20]. Similarly, we noted pronounced annual variation in the proportion of test-positive tests (4.8–14%). One of these variations (2017) was consistent with an anecdotally reported outbreak of canine leptospirosis in the Halifax region of Nova Scotia. Additionally, we noted marked variation in the canine leptospirosis test-positive proportion across the Canadian regions [gradient from 18.5% (Nova Scotia) and 9.6% (Ontario) to 1.0% (prairie provinces)]. These regional variations may reflect “hot spots” for canine leptospirosis with consistently elevated disease risk and locations likely to experience future elevated risks. However, regional variation in clinician awareness and testing patterns (e.g., only test dogs with a high suspicion for leptospirosis, test dogs along the continuum of suspicion) may also be responsible for these variations.

Multiple factors are considered to influence test-positive prevalence of canine leptospirosis [1, 2, 11, 23–25]. These reported factors have included dog location (i.e., urban vs. rural), month of testing, monthly rainfall at time of testing, and use of prevention strategies (e.g., increased vaccination efforts). The Canadian provinces with the highest test-positive proportion for canine leptospirosis in our study were Ontario and Nova Scotia. Similarly, developed case maps visualized likely areas of leptospirosis ‘hot spots’ in these two regions (high number of cases and high number of cases per human capita in given FSAs). These findings align with previous studies from the United States that observed clusters of cases and increased seroprevalence/test-positive proportion in specific regions [3, 11, 20, 26, 27]. These US-based studies have indicated that increased rainfall, flooding, and proximity to bodies of water in these regions, along with the presence of reservoir hosts could explain the observed regional distribution of canine leptospirosis. It is likely that similar environmental factors are associated with (perhaps responsible for) the noted Canadian distribution we observed; however, further investigation is needed to confirm this.

Similar to other studies [1, 20, 23, 28], we observed dogs from urban areas were at increased odds for testing positive for *Leptospira* as compared to those from rural regions. This could be due to encroaching wildlife populations, or other factors such as veterinary healthcare seeking behaviors or socioeconomic status, leading to exposure to area wildlife or domestic cats, which may act as *Leptospira* carrying reservoir hosts in these regions [29]. These urban wildlife (e.g., rodents, raccoons) and feline reservoirs have been identified as purported risk factors for canine leptospirosis [24, 30–32]. Further work identifying the regional distribution of serovars and serovar-reservoir relationships, perhaps targeting wildlife and feline reservoirs, would be useful to further guide prevention efforts, potentially including vaccine development.

Risk factor evaluation in our work shared similarities with the recent US-based study evaluating *Leptospira* PCR data [20]. Significant predictors of a positive leptospirosis test were younger age and male sex. Male sex has been repeatedly identified as a risk factor for canine leptospirosis, as demonstrated in a recent systematic review/meta-analysis [28]. Our current work adds to this ‘higher risk canine profile’ toy and terrier breeds, a finding suggested in a previous study (i.e., dogs weighing <15 pounds (6.8 kg) had the greatest odds of being diagnosed with leptospirosis) [5]. Further work examining vaccine coverage in smaller dogs in Canada, especially from urban centers, would be useful to determine if lower vaccination coverage may be playing a role in leptospirosis risk in these breeds. Historically, there have been concerns with increased adverse events in these breeds following leptospirosis vaccination and while recent data suggest these fears are largely unfounded with the current canine leptospirosis vaccines, anecdotally concerns persist [33].

Seasonal variation in canine leptospirosis has been observed in prior studies, with an increase in prevalence/proportion test-positive from late summer to early fall [9]. Potential explanations for such seasonal variations include changes in precipitation or temperature that impact survival of *Leptospira*, or seasonal canine activities or *Leptospira* reservoir host behaviors/movements that increase exposure risk for dogs [8, 20, 34]. This seasonal effect was observed in our work; we noted a trend that dogs were more likely to be test-positive September through December in zones with a greater seasonal temperature variation (e.g., Quebec and Ontario) as opposed to those without this variation (e.g., British Columbia). This finding is consistent with the recent US-based PCR study [20], but contrasts with previous MAT-based work [2, 8].

Similar to other observational studies of this type, there are limitations inherent to our work. Leptospirosis testing was performed based on clinician-owner decision, the result of which may have introduced various biases, including temporal, regional, and canine signalment-related testing approaches. Signalment data was provided by testing veterinarians or support staff, which may include data entry errors as well as potential biases for documentation varying with breed listed in the data (e.g., listed as a single breed when in fact mixed breed). Another limitation is our data were acquired from a single commercial laboratory, possibly leading to regional under-representation, and thus they might not be representative of the population as a whole. This could lead to locations for which canine leptospirosis testing data were not available (e.g., few or no test results in certain regions of Canada), resulting in regions with unmeasured *Leptospira* occurrence and a potential lack of generalization of our work. However, it is likely that these regions are of limited consequence to the overall conclusions of our work due to the historic and widespread sample submission coverage of the country (as observed by FSA test submissions, especially considering human and dog distribution in the country). Further, maps were created to provide estimates of risk levels of canine leptospirosis across Canada. These estimates are subject to various errors and biases including likely changes in test use/availability over the study period and regional and temporal differences in at-risk canine populations. Another limitation of the dataset and other studies of this type is the lack of dog clinical data and recent travel history. We assumed that samples were received from dogs presenting to veterinary practices with clinical disease consistent with leptospirosis and from an exposure relatively close to the submitting veterinary practice.

In conclusion, this work identified focal regions of canine leptospirosis in Canada, with the highest test-positive proportion (and related hot spots) in Ontario and Nova Scotia. The case maps and identified risk factors will allow practitioners and dog owners to identify areas of high risk for leptospirosis exposure and occurrence where dogs live, visit, and perform, which will allow for targeted prevention efforts.

## Supporting information

S1 File Study’s minimal data set. (CSV)

## References

[1] Alton GD, Berke O, Reid-Smith R, Ojkic D, Prescott JF. Increase in seroprevalence of canine leptospirosis and its risk factors, Ontario 1998–2006. Can J Vet Res. 2009;73(3):167–75. 19794888 PMC2705070

[2] Sykes JE, Hartmann K, Lunn KF, Moore GE, Stoddard RA, Goldstein RE. 2010 ACVIM Small animal consensus statement on leptospirosis: Diagnosis, epidemiology, treatment, and prevention. J Vet Intern Med. 2011;25:1–13. doi: 10.1111/j.1939-1676.2010.0654.x 21155890 PMC3040842

[3] Gautam R, Guptill LF, Wu CC, Potter A, Moore GE. Spatial and spatio-temporal clustering of overall and serovar-specific *Leptospira* microscopic agglutination test (MAT) seropositivity among dogs in the United States from 2000 through 2007. Prev Vet Med. 2010;96(1–2):122–31. doi: 10.1016/j.prevetmed.2010.05.017 20580454

[4] Lee HS, Levine M, Guptill-Yoran C, Johnson AJ, von Kamecke P, Moore GE. Regional and temporal variations of *Leptospira* seropositivity in dogs in the United States, 2000–2010. J Vet Intern Med. 2014;28(3):779–88. doi: 10.1111/jvim.12335 24597659 PMC4895461

[5] Lee HS, Guptill L, Johnson AJ, Moore GE. Signalment changes in canine leptospirosis between 1970 and 2009. J Vet Intern Med. 2014;28(2):294–9. doi: 10.1111/jvim.12273 24372922 PMC4857991

[6] Schuller S, Francey T, Hartmann K, Hugonnard M, Kohn B, Nally JE, et al. European consensus statement on leptospirosis in dogs and cats. J Small Anim Pract. 2015;56(3):159–79. doi: 10.1111/jsap.12328 25754092

[7] Hennenfent A, DelVento V, Davies-Cole J, Johnson-Clarke F. Expanding veterinary biosurveillance in Washington, DC: The creation and utilization of an electronic-based online veterinary surveillance system. Prev Vet Med. 2017;138:70–8. doi: 10.1016/j.prevetmed.2017.01.009 28237237

[8] Prescott JF, McEwen B, Taylor J, Woods JP, Abrams-Ogg A, Wilcock B. Resurgence of leptospirosis in dogs in Ontario: Recent findings. Can Vet J. 2002;43(12):955–61. 12561690 PMC339917

[9] Ward MP. Seasonality of canine leptospirosis in the United States and Canada and its association with rainfall. Prev Vet Med. 2002;56(3):203–13. doi: 10.1016/s0167-5877(02)00183-6 12441236

[10] Leptospirosis outbreak concerns linger for N.S. pets and owners. CTV News. 2017 Nov 6 [Cited 2022 May 30]. Available from: https://atlantic.ctvnews.ca/leptospirosis-outbreak-concerns-linger-for-n-s-pets-and-owners-1.3663429

[11] White AM, Zambrana-Torrelio C, Allen T, Rostal MK, Wright AK, Ball EC, et al. Hotspots of canine leptospirosis in the United States of America. Vet J. 2017;222:29–35. doi: 10.1016/j.tvjl.2017.02.009 28410673

[12] Lizer J, Velineni S, Weber A, Krecic M, Meeus P. Evaluation of 3 serological tests for early detection of *Leptospira*-specific antibodies in experimentally infected dogs. J Vet Intern Med. 2018;32(1):201–7. doi: 10.1111/jvim.14865 29131400 PMC5787205

[13] Day MJ, Horzinek MC, Schultz RD, Squires RA. WSAVA Guidelines for the vaccination of dogs and cats. J Small Anim Pract. 2016;57(1):E1–E45. doi: 10.1111/jsap.2_12431 26780857 PMC7166872

[14] Leutenegger CM, Palaniappan R, Elsemore D. Analytical sensitivity and specificity of a real-time PCR assay detecting pathogenic *Leptospira* in dogs based on the Hap-1 gene. J Vet Intern Med. 2009;23:772.

[15] Branger C, Blanchard B, Fillonneau C, Suard I, Aviat F, Chevallier B, et al. Polymerase chain reaction assay specific for pathogenic *Leptospira* based on the gene hap1 encoding the hemolysis-associated protein-1. FEMS Microbiol Lett. 2005 Feb 15;243(2):437–45. doi: 10.1016/j.femsle.2005.01.007 15686847

[16] Governement of Canada. Forward Sortation Area—Definition. 2015 May 5 [cited 30 May 2022]. In: Statistics and Research [Internet]. Canada. Available from: https://www.ic.gc.ca/eic/site/bsf-osb.nsf/eng/br03396.html

[17] American Kennel Club. List of breeds by group. 2020 June 3 [cited 30 May 2022]. In: General tips and information [Internet]. USA. Available from: https://www.akc.org/public-education/resources/general-tips-information/dog-breeds-sorted-groups/

[18] Statitiscs Canada. Population and dwelling count highlight tables, 2016 census. 2018 February 07 [cited 30 May 2022]. In: Population and dwelling counts [Internet]. Canada. Available from: https://www12.statcan.gc.ca/census-recensement/2016/dp-pd/hlt-fst/pd-pl/Table.cfm?Lang=Eng&T=1201&S=22&O=A

[19] Census Forward Sortation Area Boundary File, 2016 Census. Statistics Canada Catalogue no. 92-179-X.

[20] Smith AM, Arruda AG, Evason MD, Weese JS, Wittum TE, Szlosek D, et al. A cross-sectional study of environmental, dog, and human-related risk factors for positive canine leptospirosis PCR test results in the United States, 2009 to 2016. BMC Vet Res. 2019;15(1):412. doi: 10.1186/s12917-019-2148-6 31730465 PMC6858729

[21] Reagan KL, Sykes JE. Diagnosis of canine leptospirosis. Vet Clin North Am Small Anim Pract. 2019;49(4):719–31. doi: 10.1016/j.cvsm.2019.02.008 30961998

[22] Harkin KR, Roshto YM, Sullivan JT, Purvis TJ, Chengappa MM. Comparison of polymerase chain reaction assay, bacteriologic culture, and serologic testing in assessment of prevalence of urinary shedding of leptospires in dogs. J Am Vet Med Assoc. 2003;222(9):1230–3. doi: 10.2460/javma.2003.222.1230 12725310

[23] Raghavan RK, Brenner KM, Higgins JJ, Shawn Hutchinson JM, Harkin KR. Neighborhood-level socioeconomic and urban land use risk factors of canine leptospirosis: 94 cases (2002–2009). Prev Vet Med. 2012;106(3–4):324–31. doi: 10.1016/j.prevetmed.2012.04.003 22626864

[24] Hennebelle JH, Sykes JE, Foley J. Risk factors associated with leptospirosis in dogs from Northern California: 2001–2010. Vector Borne Zoonotic Dis. 2014;14(10):733–9. doi: 10.1089/vbz.2014.1624 25325317

[25] Francey T, Schweighauser A, Reber A, Schuller S. Evaluation of changes in the epidemiology of leptospirosis in dogs after introduction of a quadrivalent antileptospiral vaccine in a highly endemic area. J Vet Intern Med. 2020;34(6):2405–17. doi: 10.1111/jvim.15947 33103800 PMC7694862

[26] Moore GE, Guptill LF, Glickman NW, Caldanaro RJ, Aucoin D, Glickman LT. Canine leptospirosis, United States, 2002–2004. Emerg Infect Dis. 2006;12(3):501–3. doi: 10.3201/eid1203.050809 16704794 PMC3291439

[27] Smith AM, Stull JW, Evason MD, Weese JS, Wittum TE, Szlosek D, et al. Investigation of spatio-temporal clusters of positive leptospirosis polymerase chain reaction test results in dogs in the United States, 2009 to 2016. J Vet Intern Med. 2021;35(3):1355–60. doi: 10.1111/jvim.16060 33729616 PMC8163129

[28] Ricardo T, Previtali MA, Signorini M. Meta-analysis of risk factors for canine leptospirosis. Prev Vet Med. 2020;181:105037. doi: 10.1016/j.prevetmed.2020.105037 32590226

[29] Murray MH, Fidino M, Fyffe R, Byers KA, Pettengill JB, Sondgeroth KS, et al. City sanitation and socioeconomics predict rat zoonotic infection across diverse neighbourhoods. Zoonoses Public Health. 2020;67(6):673–83. doi: 10.1111/zph.12748 32583624

[30] Ghneim GS, Viers JH, Chomel BB, Kass PH, Descollonges DA, Johnson ML. Use of a case-control study and geographic information systems to determine environmental and demographic risk factors for canine leptospirosis. Vet Res. 2007;38(1):37–50. doi: 10.1051/vetres:2006043 17074294

[31] Murillo A, Goris M, Ahmed A, Cuenca R, Pastor J. Leptospirosis in cats: Current literature review to guide diagnosis and management. J Feline Med Surg. 2020;22(3):216–28. doi: 10.1177/1098612X20903601 32093581 PMC11132596

[32] Dorsch R, Ojeda J, Salgado M, Monti G, Collado B, Tomckowiack C, et al. Cats shedding pathogenic *Leptospira* spp.-An underestimated zoonotic risk? PLoS One. 2020;15(10):e0239991. doi: 10.1371/journal.pone.0239991 33091006 PMC7580889

[33] Yao PJ, Stephenson N, Foley JE, Toussieng CR, Farver TB, Sykes JE, et al. Incidence rates and risk factors for owner-reported adverse events following vaccination of dogs that did or did not receive a *Leptospira* vaccine. J Am Vet Med Assoc. 2015;247(10):1139–45. doi: 10.2460/javma.247.10.1139 26517617

[34] Ward MP, Guptill LF, Prahl A, Wu CC. Serovar-specific prevalence and risk factors for leptospirosis among dogs: 90 cases (1997–2002). 10.2460/javma.2004.224.1958 15230451

